# Bovine Induced Pluripotent Stem Cells Are More Resistant to Apoptosis than Testicular Cells in Response to Mono-(2-ethylhexyl) Phthalate

**DOI:** 10.3390/ijms15035011

**Published:** 2014-03-20

**Authors:** Ying-Chu Lin, Kung-Kai Kuo, Kenly Wuputra, Shih-Han Lin, Chia-Chen Ku, Ya-Han Yang, Shin-Wei Wang, Sheng-Wen Wang, Deng-Chyang Wu, Chun-Chien Wu, Chee-Yin Chai, Cheng-Lung Lin, Chang-Shen Lin, Masayuki Kajitani, Hiroyuki Miyoshi, Yukio Nakamura, Shinichi Hashimoto, Kouji Matsushima, Chunyuan Jin, Shau-Ku Huang, Shigeo Saito, Kazunari K. Yokoyama

**Affiliations:** 1 School of Dentistry, Kaohsiung Medical University, Kaohsiung 807, Taiwan; 2 Cancer Center, Kaohsiung Medical University and Kaohsiung Medical University Hospital, Kaohsiung 807, Taiwan; E-Mail: chulin@cc.kmu.edu.tw; 3 College of Medicine, Kaohsiung Medical University and Kaohsiung Medical University Hospital, Kaohsiung 807, Taiwan; E-Mails: kuoksfo@yahoo.com.tw (K.-K.K.); sal9522059@yahoo.com.tw (Y.-H.Y.); swang910@gmail.com (S.-W.W.); dechwu@yahoo.com (D.-C.W.); lazzz.wu@gmail.com (C.-C.W.); cychai@kmu.edu.tw (C.-Y.C.); 4 Graduate Institute of Medicine, Kaohsiung Medical University, Kaohsiung 807, Taiwan; E-Mails: kenlywu@hotmail.com (K.W.); korosakihisoka@yahoo.com.tw (S.-H.L.); r991046@gap.kmu.edu.tw (C.-C.K.); silviaw@hotmail.com.tw (S.-W.W.); Stevelin@kmu.edu.tw (C.-L.L.); changshen.lin@gmail.com (C.-S.L.); 5 Department of Environmental Medicine, NYU School of Medicine, Tuxedo, NY 10987, USA; E-Mail: Chunyuan.Jin@nyumc.org; 6 School of Science and Engineering, Teikyo University, Utsunomiya, Tochigi 329-2192, Japan; E-Mail: kajitani@nasu.bio.teikyo-u.ac.jp; 7 RIKEN BioResource Center, Tsukuba, Ibaraki 305-0074, Japan; E-Mails: miyoshi@brc.riken.jp (H.M.); yukionak@brc.riken.jp (Y.N.); 8 Department of Molecular Preventive Medicine, Graduate School of Medicine, the University of Tokyo, Tokyo 113-8655, Japan; E-Mails: hashimot@m.u-tokyo.ac.jp (S.H.); koujim@m.u-tokyo.ac.jp (K.M.); 9 Division of Environmental Health and Occupational Medicine, National Health Research Institutes, 35 Keyan Rd, Zhunan, Miaoli County 350, Taiwan; E-Mail: skhuang1@gmail.com; 10 Saito Laboratory of Cell Technology, Yaita, Tochigi 329-2192, Japan

**Keywords:** bovine iPSCs, testicular cells, OCT4, electroporation, endocrine disruptor, frizzled receptor, WNT signal, androgen receptor

## Abstract

Although the androgen receptor (AR) has been implicated in the promotion of apoptosis in testicular cells (TSCs), the molecular pathway underlying AR-mediated apoptosis and its sensitivity to environmental hormones in TSCs and induced pluripotent stem cells (iPSCs) remain unclear. We generated the iPSCs from bovine TSCs via the electroporation of OCT4. The established iPSCs were supplemented with leukemia inhibitory factor and bone morphogenetic protein 4 to maintain and stabilize the expression of stemness genes and their pluripotency. Apoptosis signaling was assessed after exposure to mono-(2-ethylhexyl) phthalate (MEHP), the active metabolite of di-(2-ethylhexyl) phthalate. Here, we report that iPSCs were more resistant to MEHP-induced apoptosis than were original TSCs. MEHP also repressed the expression of AR and inactivated WNT signaling, and then led to the commitment of cells to apoptosis via the cyclin dependent kinase inhibitor p21^CIP1^. The loss of the frizzed receptor 7 and the gain of p21^CIP^ were responsible for the stimulatory effect of MEHP on AR-mediated apoptosis. Our results suggest that testicular iPSCs can be used to study the signaling pathways involved in the response to environmental disruptors, and to assess the toxicity of environmental endocrine disruptors in terms of the maintenance of stemness and pluripotency.

## 1. Introduction

Phthalates are synthetic compounds that are used widely as plasticizers, solvents, and additives in many consumer products, and are known as a group of endocrine-disrupting chemicals (EDCs) that affect the male reproductive system, as assessed in animal studies. Several previous studies have reported that the major cellular targets of phthalates in the male reproductive organs are the Sertoli and Leydig cells of the testis [1,2]. To date, many studies have confirmed that exposure to mono-(2-ethylhexyl) phthalate (MEHP) in male leads to testicular atrophy and decreases testicular weight and testosterone levels [2]. Sertoli cells are the primary target of di-(2-ethylhexyl) phthalate (DEHP) and its active metabolite MEHP in rodents, with peripubertal animals being particularly sensitive to phthalate-induced injury [3]. Phthalate-induced injury to Sertoli cells reduces their ability to support germ-cell development [1]. Many reports have documented the detachment of germ cells from the seminiferous epithelium and the increase in the incidence of germ cell apoptosis in young peripubertal rodents after exposure to MEHP [4]. These phthalates’ exposure reduces the number of germ cells in the fetal mouse testis via androgen receptor (AR)- and estrogen-receptor-independent mechanisms [5]. Both DEHP and MEHP cause oxidative DNA damage in testis by inducing apoptosis in testicular cells [6]. The exposure of perinatal or young adult rodents to phthalates, including MEHP, also reduces their sperm counts [7]. Specifically, after exposure to 10^−4^ M MEHP for three days, the number of germ cells in cultured human fetal testes is reduced by 40%, resulting from a large increase in apoptosis [8]. Recently, Muczynski *et al*. [9] reported that exposure to 10^−5^ M MEHP reduces the *in vivo* development of the human fetal male germ cells. However, the direct effects of MEHP on apoptosis in embryonic stem cells (ESCs) and induced pluripotent stem cells (iPSCs) remain unclear.

iPSCs have been generated from somatic cells by the addition of several combinations of transcription factors (OCT4, c-MYC, KLF4, and SOX2) [10]. These reprogramming factors produce ESC-like morphologies and functionalities in cells by activating pluripotency-associated genes, and by repressing differentiation-promoting genes. The maintenance of a pluripotent state in ESCs depends on key molecular signaling pathways. The leukemia inhibitory factor (LIF) has been identified as an important mediator that supports the maintenance of pluripotency in murine ESCs via the Stat3 pathway [11]. ESCs can be propagated in medium containing the bone morphogenetic protein 4 (BMP4) in the absence of feeder cells and serum. It has been suggested that the same pathways influence the generation and maintenance of both ESCs and iPSCs [12]. Human ESCs and iPSCs were recently converted to the naïve pluripotent state by propagating the cells in LIF, together with the addition of inhibitors of ERK1/2 and glycogen synthase kinase-3, such as PD98059 or CHIR99021, to the medium [12,13].

The WNT signaling pathway is known to be involved in cell differentiation, migration, and proliferation during embryonic development [14]. The Frizzled (FZD) receptor responded to WNT proteins in the presence of the WNT corepressor to activate the canonical and noncanonical WNT pathways. Among the FZD family, FZD7 played an important role in maintaining stem cells in an undifferentiated state [15]. However, the effects of MEHP exposure on these signaling pathways in ESCs and iPSCs remain unsolved.

In this study, we generated bovine iPSCs from testicular cells via the electroporation of OCT4. We report the effects of exposure to the environmental endocrine-disrupting phthalate metabolite, MEHP, on AR-mediated apoptosis and WNT/Frizzled signaling in testicular cells and testicular iPSCs. We also examined the global impact of MEHP on the molecular signaling cascade that underlies AR-dependent apoptosis and unveiled the molecular target of MEHP to understand its mechanism of toxicity in iPSCs. The results of this study will be useful for regenerative-medicine approaches that use adult testicular stem cells or iPSCs, understand the toxicological effects of ESCs, and provide a model system for the creation of ESC-based therapeutic agents for damaged testicular tissues.

## 2. Results

### 2.1. Generation of iPSCs from Bovine Testis Cells

The voltage intensities used for electroporation were evaluated to optimize the efficiency of the transfection of bovine testicular cells with the enhanced green fluorescent protein expression vector (pEGFP). As shown in Figure S1, electroporation using 10 electrical pulses of 20 V at 50 ms intervals was required for the efficient transfection of bovine testicular cells. This yielded the highest survival rate and transfection frequency (63.3% and 66.7%, respectively; see Table S1). After three passages (15–21 days) of the testicular cells without feeder cells, we obtained compact, elliptical colonies that expressed pluripotency marker genes, such as *KLF4*, *c-MYC*, *STAT3*, *DNMT1*, *SUZ12*, and *MEF2A*, and did not express *OCT4*, and *SOX2*, or *NANOG* (data not shown).

**Figure 1.**
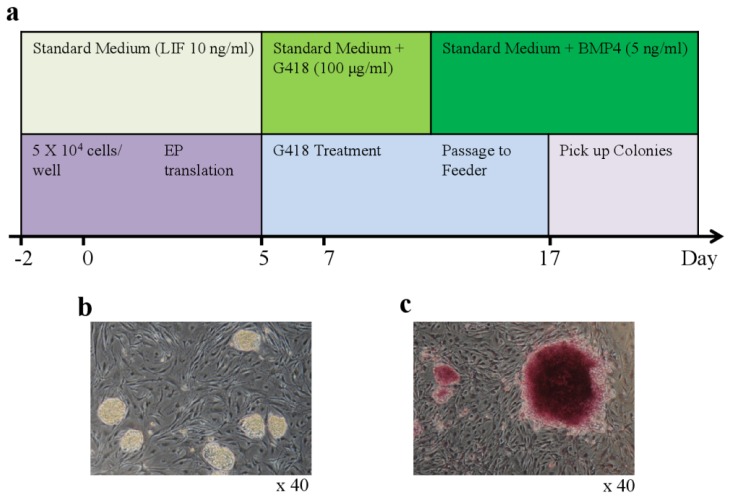
Generation of iPSCs from bovine testicular cells. (**a**) Schematic diagram of the generation of bovine iPSCs by electroporation-mediated transfection; (**b**) Typical morphology of bovine iPSC colonies generated with the single factor OCT4 on day 25 after electroporation (magnification ×40); (**c**) Alkaline phosphatase staining of bovine iPSCs in large colonies of fibroblasts (magnification ×40).

Subsequently, bovine testicular cells were transfected by electroporation with a plasmid encoding OCT4. Small, packed, and domed colonies were detected on mitotically inactivated MEFs 17 days after transfection (Figure 1a–c). These colonies were composed of small and rapidly dividing cells with a high nuclear/cytoplasmic ratio and large nucleoli. The estimated reprogramming efficiency was 0.3%, which was 20-fold higher than the efficiency of the one-factor (1F) approach that has been used to reprogram murine neural stem cells [16–18]. After colonies were picked manually, the bovine iPSCs were passaged. The number of colonies with the typical iPSC phenotype increased over time and by repeated passaging (Figure 1b). From the initial input of 5 × 10^4^ cells, we obtained 10 testicular colonies at the second passage, from which we eventually obtained approximately 20 iPSC colonies. Subculture of iPSCs for more than four weeks under conditions that were specific for bovine iPSCs yielded cells with a strong alkaline phosphatase activity (Figure 1c). Immunofluorescence staining confirmed expression of endogenous “stemness” markers, including OCT4, NANOG, SOX2, SSEA-1, and SSEA-4, in bovine 1F iPSCs (Table 1). These markers were most intense in dense patches of cells with the typical 1F iPSC morphology. The G-banding karyotype analysis of metaphase spreads revealed normal distributions of the 60 chromosomes, including the X and Y sex chromosomes, at passage 15 (data not shown). Transcription of the *OCT4*, *SOX2*, *c-MYC*, *KLF4*, *MEF2A*, *SUZ12*, *STAT3*, and *DNMT1* genes was also detected in the bovine 1F iPSCs (Table 1).

### 2.2. Pluripotency of 1F-iPSCs

**Figure 2.**
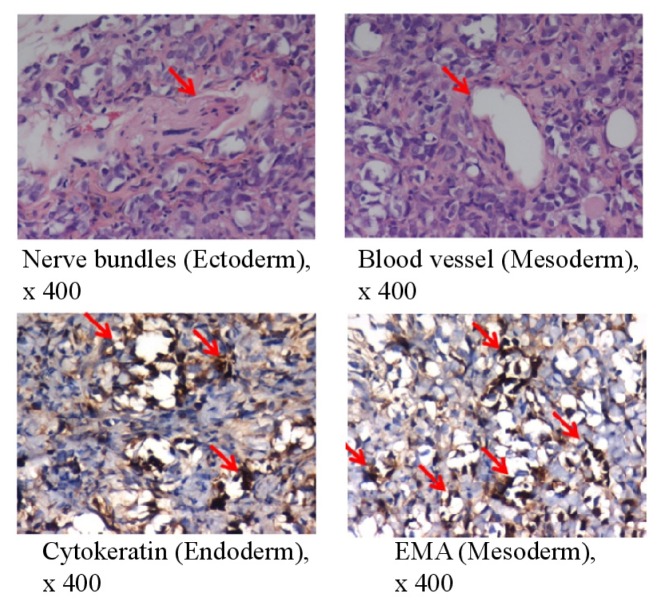
*In vivo* pluripotency of bovine iPSCs. Teratoma formation at 6–8 weeks after the transplantation of 1F bovine iPSCs into SCID mice was examined. The teratomas were sectioned and stained with hematoxylin and eosin 6–8 weeks after transplantation (**upper panels**); The sections were stained with antibodies specific for cytokeratin (epithelial cells) or EMA (epithelial cells) (**lower panels**). Magnification ×400. The red arrow indicates nerve bundles, blood vessels, cytokeratin, and EMA, respectively.

To assess the pluripotency of bovine 1F iPSCs *in vivo*, we transferred the cells into immunodeficient SCID mice. The bovine iPSCs generated benign cystic teratomas containing mature tissues of all three germ layers. Immunohistochemical staining for neural bundles and blood vessels indicated ectoderm and mesoderm differentiation, respectively. The epithelial membrane antigen (EMA; also called MUC1), which is a marker of endodermal differentiation, was also detected (Figure 2). The proliferation index of whole teratomas was <3%.

### 2.3. Effects of the Phthalate Ester MEHP on Apoptosis

**Figure 3.**
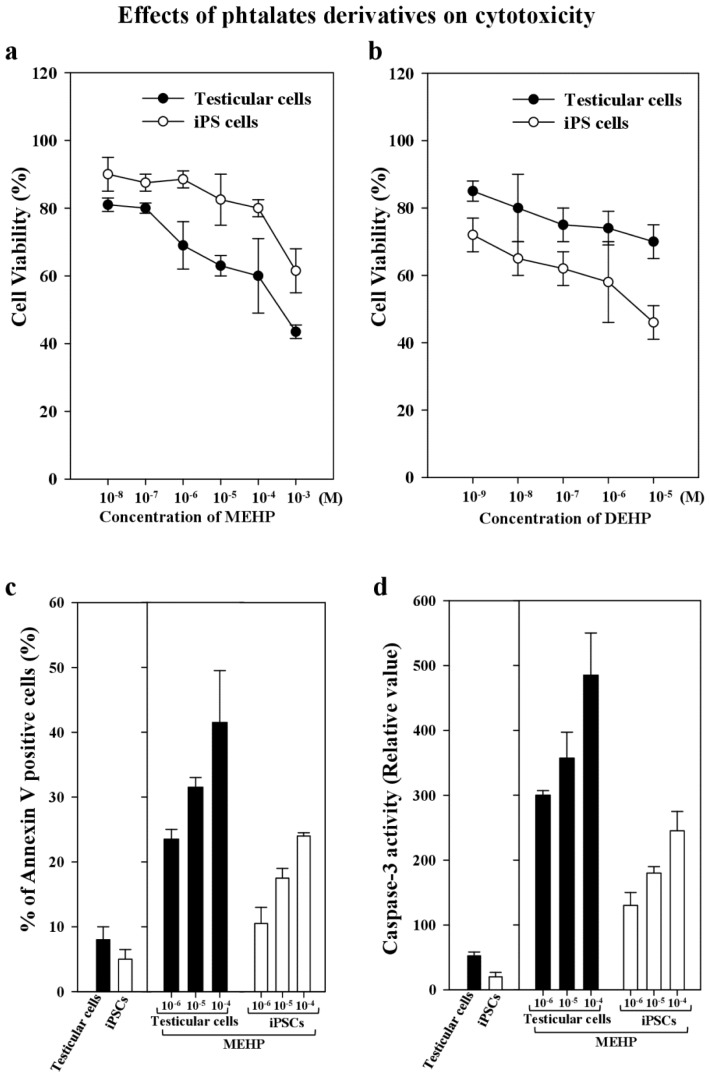
Cell viability and apoptosis induced by phthalate derivatives in bovine testicular cells and iPSCs. (**a**) Cell viability was measured by Trypan Blue staining in the presence or absence of phthalate esters for 24 h; (**b**) Cell viability was measured by Trypan Blue staining in the presence or absence of DEHP for 24 h; (**c**) Apoptotic activity was measured by FITC-labeled annexin V, followed by flow cytometric analysis to identify the apoptotic cells, as described in the Experimental Section; (**d**) Caspase-3 activity in the testicular cells and iPSCs was measured after 10^−4^–10^−6^ M MEHP and 10^−7^ M staurosporin as a control were added to them for 24 h; and their apoptotic activity was analyzed. Data presented are means ± SD. The statistical analysis was performed with Student’s *t*-test.

DEHP is metabolized to its monoester metabolite, MEHP, which is formed when DEHP is rapidly hydrolyzed by lipases in the gut. Thus, it becomes an active testicular toxicant that inhibits the androgenic response indirectly [7,19,20]. Therefore, we compared the stability of stemness gene expression, the differentiation potency, and the apoptosis of bovine testicular cells and corresponding iPSCs after exposure to MEHP. We found that the viability of testicular cells was 80% less than that of testicular iPSCs after exposure to MEHP or DEHP (Figure 3a,b). In contrast, the apoptotic activity of annexin-V-stained cells or the caspase 3 activity of testicular cells exposed to 10^−4^ to 10^−6^ M MEHP was significantly higher than that of iPSCs (1.75–2.1-fold; Figure 3c,d).

### 2.4. Regulation of AR, p21^CIP1^, and Apoptosis

**Figure 4.**
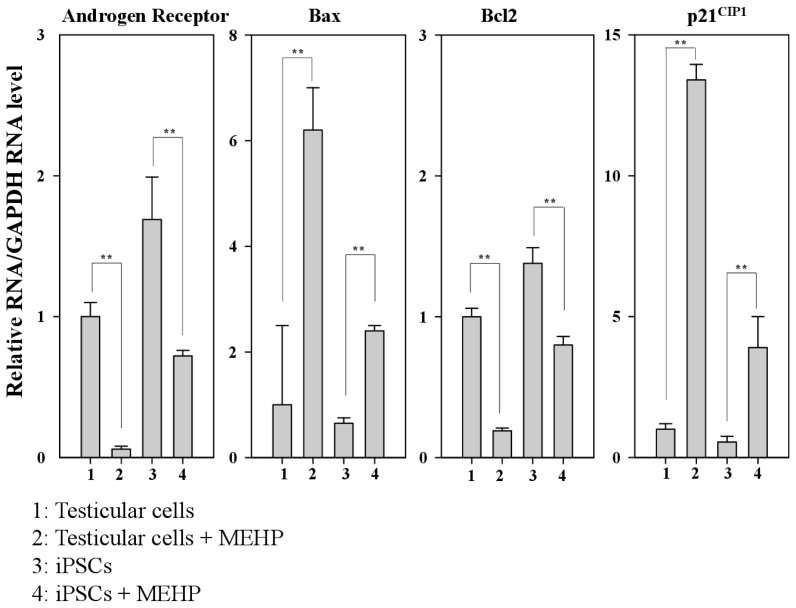
Relative expression of genes related to androgen-receptor-mediated apoptotic signaling. Real-time PCR was performed using the bovine-specific primers listed in Table 2. Relative expression of the genes encoding the AR, BAX, BCL-2 and p21^CIP1^ are shown in bovine testicular cells and iPSCs. Values indicated are means ± SEM, *n* ≥ 3; ^**^ *p* < 0.01.

Previous studies have reported the role of AR in the regulation of apoptosis in prostate cancer [20–22]. Moreover, AR-mediated apoptosis is inhibited by the increased expression of the cyclin dependent kinase inhibitor p21^CIP1^ or the phosphorylation of AR induced by AKT or MAPK [23–25]. Therefore, we designed bovine-specific primers to detect the bovine AR mRNA and avoid the noise arising from contamination with MEF feeder cells. The expression of AR in testicular cells was reduced by 60% compared with that observed in iPSCs. We also found that MEHP significantly reduced the expression of AR in testicular cells compared with that detected in iPSCs (reduction of 90% *vs*. 60%, respectively) (Figure 4). However, after treatment with MEHP, the expression of the apoptosis-related gene BAX was increased by 6.2-fold in testicular cells and by 3.7-fold in iPSCs compared with the control. In contrast, the expression of the anti-apoptotic gene BCL-2 was reduced by 80% in testicular cells and by 50% in iPSCs, after incubation of the cells with MEHP. In the absence of MEHP, the expression of BAX declined by 65%, whereas BCL2 expression increased by 1.6-fold in iPSCs. Furthermore, the expression of p21^CIP1^ increased by 13.5-fold in testicular cells and by 7.2-fold in iPSCs, when both types of cells were treated with MEHP. Although the expression of p21^CIP1^ in iPSCs was only 55% of that detected in testicular cells, MEHP enhanced the expression of p21^CIP1^ in testicular cells to a greater extent than it did in iPSCs. It was reported that a MEHP-mediated increase in p21^CIP1^ may be involved in AR-dependent apoptosis [23–25]. Thus, we propose that the expression of p21^CIP1^ is critical for AR-mediated apoptosis in testicular cells.

### 2.5. Role of p21^CIP1^ in Phthalate-Induced Apoptosis

**Figure 5.**
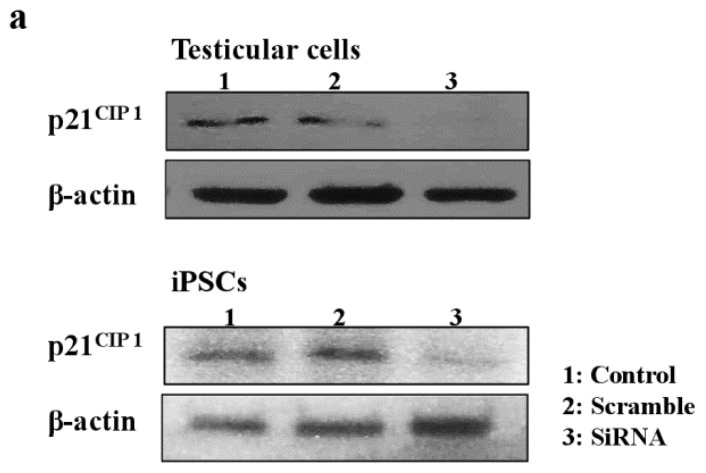

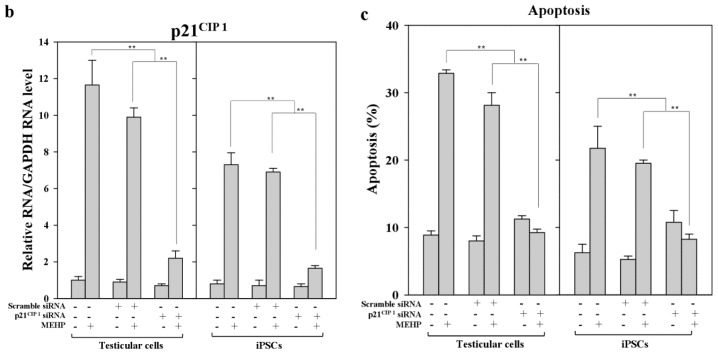
Effects of the knockdown of p21^CIP1^ expression by siRNA on gene expression and apoptosis. Bovine iPSCs were treated with p21^CIP1^ siRNA, or a scrambled nonspecific siRNA, and treated with DMSO or 10^−4^ M MEHP for 12 h. (**a**) The expression levels of p21^CIP1^ were measured by Western blotting as described in the Experimental section. Lane 1; non-treated siRNA; lane 2, scramble siRNA; lane 3, p21^CIP1^ specific siRNA; (**b**) The expression levels of p21^CIP1^ were measured in triplicate samples by qRT-PCR, and were corrected to the GAPDH RNA levels; (**c**) Apoptotic cells were quantified by staining with annexin V, as described in the Experimental Section. (−, control DMSO (0.001%); +, 10^−4^ M MEHP in the presence (+) or absence (−) of the p21^CIP1^siRNA or scrambled siRNA). The values expressed as means ± SEM, *n* ≥ 3; ^**^ *p* < 0.01.

Next, we examined the effects of MEHP on the expression of AR and p21^CIP1^ in iPSCs, and found that AR levels were repressed by exposure to the phthalate MEHP. In contrast, treatment with phthalates increased the level of the p21^CIP1^ protein in iPSCs, but not in MEFs [19]. The RNA levels in MEHP showed similar results (Figure 5). To understand the link between MEHP-mediated AR repression and apoptosis induction, we introduced a small interfering RNA (siRNA) against p21^CIP1^ in bovine testicular iPSCs; we observed that this siRNA, but not a scrambled siRNA, reduced p21^CIP1^ protein and RNA levels (Figure 5a,b), and completely attenuated MEHP-induced apoptosis (Figure 5c). These activities were more significant in testicular cells compared with iPSCs.

### 2.6. Repression of the Wnt Receptor Frizzled 7 by MEHP

**Figure 6.**
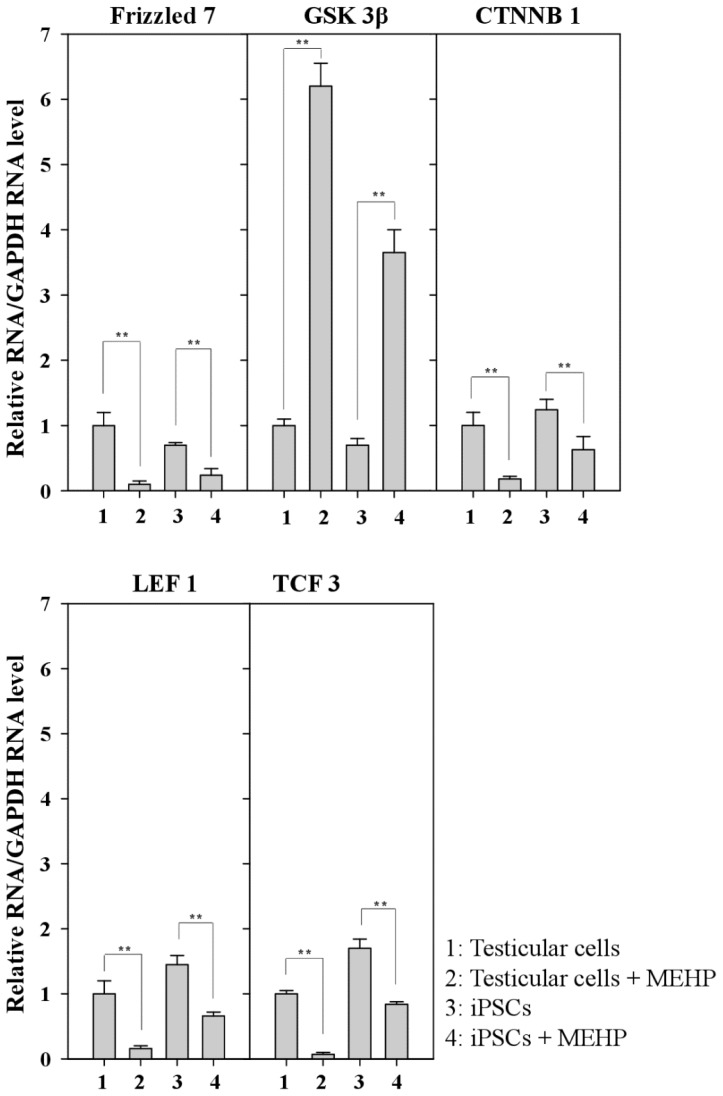
Relative expression of genes related to the WNT/β-catenin canonical pathway. Relative expression of a series of Frizzled, GSK3β, CTNNB, LEF1, and TCF3 in bovine testicular cells and iPSCs. (Lane 1, control DMSO (0.001%) in testicular cells; lane 2, 10^−4^ M MEHP in testicular cells; lane 3, control DMSO (0.001%) in iPSCs; lane 4, 10^−4^ M MEHP in iPSCs. The values expressed as means ± SEM, *n* ≥ 3; ^**^ *p* < 0.01.

AR signaling is affected by WNT/β-catenin signaling [26,27]. We examined the expression of genes related to the WNT pathway after exposure to MEHP (Figure 6). Treatment with MEHP inhibited the expression of the frizzled receptor 7 (FZD7) (Figure 6). Thus, WNT signaling was completely blocked by MHEP because its receptor, FZD7, was selectively repressed. Therefore, the expression of GSK3β was activated and the expression of β-catenin and LEF1/TCF3 was preferentially repressed (Figure 6). Taken together, these results suggest that MEHP preferentially suppresses WNT signaling by inactivating FZD7, and that the effect of MEHP is more significant in testicular cells than it is in iPSCs.

### 2.7. Effect of MEHP on WNT/Frizzled Signaling and AR-Mediated Apoptosis

**Figure 7.**
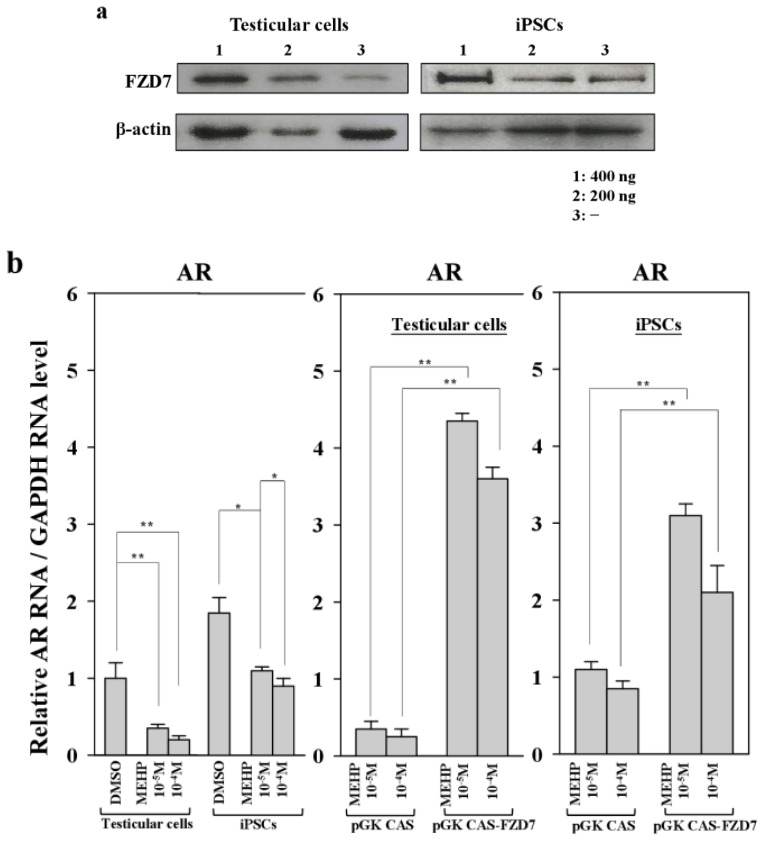

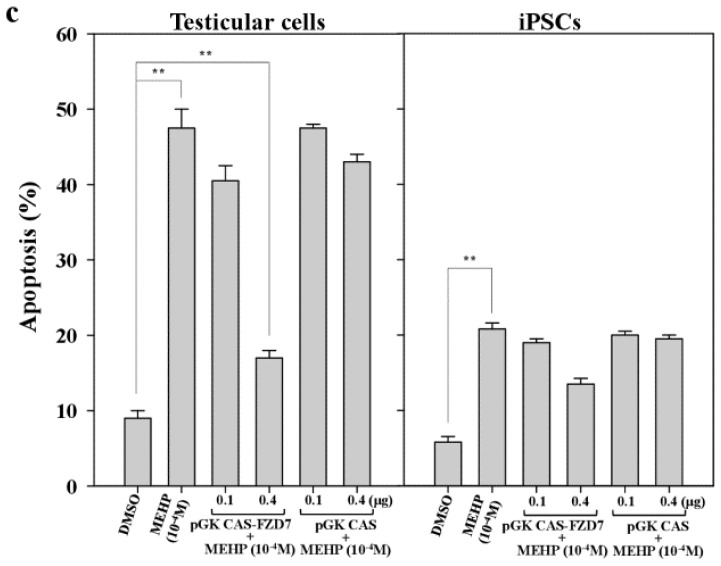
Effects of the forced expression of the Frizzled receptor FZD7 on gene expression and apoptosis in testicular cells and iPSCs. (**a**) Protein expression of FZD7 in bovine iPSCs and testicular cells transfected with pGK-CAS-FZD7 (200 and 400 ng) were examined. After cultivation of transfectants for 24 h, MEHP (10^−4^ and 10^−5^ M) were added and cultured for another 24 h and then subjected to Western blotting, as described in the Experimental Section; (**b**) pGK-CAS-FZD7 (400 ng) or each control vector pGK-CAS (400 ng) was introduced into bovine iPSCs, which were cultured for 24 h; 10^−4^ M MEHP was added and the cells cultured for another 24 h. The relative expression of *AR* was quantified by quantitative polymerase chain reaction (qPCR), as described in the Experimental section; (**c**) Effect of FZD7 on apoptosis. Apoptotic cells were quantified as described in the Experimental Section. Values measured with DMSO were defined as 1. Values indicated are means ± SEM, *n* ≥ 3; ^*^ *p* < 0.05, ^**^ *p* < 0.01.

To confirm the link between FZD7 and AR-mediated apoptosis in both testicular cells and testicular iPSCs, we introduced an expression vector encoding FZD7 and examined the expression of the *AR* gene. As shown in Figure 7a, two- to three-fold increases in FZD7 protein were detected in both testicular cells and iPSCs. The forced expression of FZD7 increased the expression of AR more efficiently in testicular cells than it did in iPSCs, at the RNA level (10–12-fold *vs.* 2.5–2.8-fold, respectively; Figure 7b). Furthermore, apoptosis was rescued by the forced expression of FZD7, even in the presence of MEHP (Figure 7c). We also examined the rescue of WNT signaling by the forced expression of FZD7. The upregulation of GSK3β and the downregulation of CTNNB1 in response to MEHP were rescued by the forced expression of FZD7 (Figure S2a–e). This effect was more evident in testicular cells than it was in iPSCs (Figure S2).

## 3. Discussion

This study has several important implications. First, it shows that electroporation was an efficient way to transfer genes into bovine testicular cells; Second, the introduction of OCT4 alone was demonstrated sufficient to induce iPSCs in the presence of LIF and BMP4; therefore, the ectopic expression of SOX2, KLF4, and c-MYC was not required; Third, the environmental EDC MHEP triggered apoptosis to a greater extent in bovine testicular cells than it did in iPSCs; Fourth, MEHP induced significant apoptosis by downregulating the expression of AR and upregulating p21^CIP1^; Fifth, MEHP blocked the WNT cascade and enhanced AR-mediated apoptosis via a decrease in the expression of FZD7.

In bovine testicular cells, we did not observe endogenous expression of OCT4, NANOG, or SOX2, but detected KLF4 and c-MYC expression. Exogenous OCT4 was reported to induce the expression of SOX2 via a transactivation mechanism, possibly through octamer-binding sites [16]. In addition, the promoter of the *NANOG* gene was stimulated by the forced expression of *OCT4* and *SOX2* in ESCs [28]. Therefore, we predicted that OCT4 itself is capable of reprogramming testicular cells via a network leading to OCT4/SOX2/NANOG expression. Moreover, although the generation of human testicular stem cells has been reported, the frequency of pluripotent stem cells is lower, approximately 1 to 10^6^ testis stem cells [29]. Thus, we decided to establish iPSCs, instead of adult stem cells, to perform further additional experiments, because of the established protocol for generation.

The generation of ESCs is particularly sensitive to the dose of OCT4. For example, a 50% increase or reduction in the level of OCT4 causes their differentiation into cells that express markers of the endoderm and mesoderm or trophectoderm, respectively [30]. Therefore, OCT4 is a critical factor in nuclear reprogramming and cellular self-renewal. The use of the fibroblast growth factor 2 (FGF2), instead of both LIF and BMP4, led to the differentiation of bovine iPSCs differentiated toward neural progenitor cells (data not shown), indicating that bovine iPSCs differ from human iPSCs, the characteristics of which include a dependence on FGF2/activin signaling, a flat morphology, and reduced tolerance of single-cell dissociation by trypsin.

The expression of pluripotency markers, including OCT4, NANOG, SOX2, STAT3, c-MYC, KLF4, TERT, and DNMT3A, was maintained in bovine iPSCs. Mouse ESCs and iPSCs express SSEA-1, but not SSEA-4, whereas human ESCs and iPSCs express SSEA-4, but not SSEA-1 [13]. The morphology and the expression of the SSEA antigens of these bovine iPSCs resembled those of mouse ESCs/iPSCs, rather than those of human ESCs/iPSCs. Pig iPSCs are also positive for SSEA-4, but not for SSEA-1, and exhibit morphologies that are similar to those of human ESCs/iPSCs [31]. Interestingly, bovine iPSCs express both SSEA-1 and SSEA-4, whereas SSEA-1 expression is observed in both equine and bovine embryonic stem-like cells, as reported previously by our group [32,33]. In addition to SSEA-1, we found a strong signal for SSEA-4, which has not been reported previously in bovine ES-like cells [17]. Therefore, our iPSCs are more similar to naïve iPSCs than they are to iPSCs derived from fibroblasts [33], suggesting that bovine testicular cells can be reprogrammed more easily than fibroblasts.

In the present study, iPSCs and testicular cells were used as *in vitro* models to evaluate the toxicity of the main metabolite of DEHP, MEHP. It has been reported that, in the testis, MEHP induces apoptosis specifically in spermatogenic cells, Sertoli cells, and organ-cultured cells from neonatal and fetal rat testes [34]. Here, we compared the sensitivity to apoptosis of testicular cells and iPSCs exposed to MEHP, and found that iPSCs were more resistant than testicular cells to MEHP-induced apoptosis.

To maintain the characteristic stemness of iPSCs, we cultured them with mitomycin-C-treated mouse fibroblast cells (MEFs), which were used as feeder cells. In the absence of MEFs, the stemness features were lost. Thus, we first screened the appropriate antibodies which detected bovine and mouse proteins in MWA high-throughput system, as described previously [19]. We recently reported that phthalate derivatives, such as DEHP, di-(*n*-butyl) phthalate, and butyl benzyl phthalate, upregulated p21^CIP1^ and BAX, and repressed AR and BCL-2 [19]. However, even in this case, we cannot exclude the interaction effects of stem cell niches, as reported previously [35]. Thus, here, we measured only bovine-specific RNA levels for comparison with MEHP treatment, to avoid the effect of mouse feeder cells. We used bovine iPSCs and their original testicular cells to examine the effects of DECs, such as MEHP, on the capacity of cells to maintain their pluripotency *in vivo* and *in vitro*. The cytotoxicity results obtained for MEHP indicate that iPSCs are more resistant to this metabolite compared with testicular cells (by about 1.3-fold; Figure 3a). This conclusion was confirmed by apoptosis assays, such as annexin V staining and caspse-3 analysis (Figure 3c,d). MEHP had a greater effect on apoptosis in testicular cells than in iPSCs (1.7- and 2.1-fold, respectively).

Previous studies have reported the role of AR in regulating apoptosis in prostate cancer [21,22]. Moreover, AR-mediated apoptosis is inhibited by the increased expression of the cyclin-dependent kinase inhibitor p21^CIP1^ or the phosphorylation of AR induced by AKT or MAPK [23–25]. Therefore, we focused on the signaling cascades that are involved in AR-mediated apoptosis, to compare the sensitivity of testicular cells with that of iPSCs. AR expression was reduced by ~80% in testicular cells and by 58% in iPSCs after MEHP exposure (Figure 4). MEHP increased apoptosis in bovine testicular cells more significantly than it did in iPSCs (Figure 3), and its AR-mediated apoptosis was regulated by p21^CIP1^ (Figures 4 and 5). This difference in the expression levels of AR and p21^CIP1^ was critical for AR-mediated apoptosis and for the WNT signaling pathway (Figure 6). Furthermore, this signaling pathway was mediated by the inactivation of the WNT receptor FZD7 after exposure to MEHP, indicating that AR-mediated apoptosis involves the activation of WNT signaling. Therefore, we speculate that bovine testicular iPSCs are more resistant to apoptosis than bovine testicular cells, which occurs via the control of p21^CIP1^ and FZD7.

To identify the signaling cascade that is involved in apoptosis and the WNT receptor FZD7, we introduced an expression vector encoding the *FZD7* gene into bovine testicular cells and the corresponding iPSCs. We found that MEHP exposure could inactivate the *FDZ7* gene expression. One possible explanation is that MEHP represses the expression of LEF/TCF, which leads to the inactivation of the *FZD7* gene. Other transcription factors, such as SP1, CAAT-binding protein, and PU.1, are not involved in MEHP-induced *FZD7* gene expression [36]. Exposure to MEHP also enhanced the expression of p53 (data not shown), which might mediate the p53–p21^CIP1^ circuit and activate p21^CIP1^ expression. Thus, MEHP may exert different effects on the signaling process that is involved in AR-mediated apoptosis in testicular cells. The elucidation of the precise mechanism underlying this phenomenon requires further experiments. In summary, we generated iPSCs from bovine testicular cells via the electroporation of the *OCT4* gene. Our data suggest that iPSCs are more resistant to AR-dependent apoptosis than are testicular cells, which occurs via the regulation of the AR–p21^CIP1^ cascade and Wnt/Frizzled signaling.

Thus, we suggest that iPSCs are useful for screening the toxicity of environmental EDCs that might affect the early embryonic development and pluripotency of stem cells in domestic animals. This screening may provide a good model to study the effects of EDCs on human development.

## 4. Experimental Section

### 4.1. Reagents and Plasmids

MEHP and DEHP were purchased from Sigma-Aldrich Chemical Co. (Milwaukee, WI, USA). The caspase 3 assay kit and bezyloxycarbonyl-Val-Ala-Asp-Ch2F (Z-VAD) were from Promega Corp. (Madison, WI, USA). The trypan blue staining solution (0.5%) was from Nacalai Tesque (Tokyo, Japan). Biotin-16-2′-deoxyuridine-5’-triphosphate, proteinase K, and the blocking reagent were from Roche Diagnostics (Mannheim, Germany). The pCMV-Flag-hOCT3/4 (RDB6598) plasmid was obtained from the RIKEN DNA Bank (Tsukuba, Ibaraki, Japan), and the pEGFP plasmid was generated as described elsewhere [19,37]. The pGK-CAS-FZD7 plasmid was a kind gift from Karl Willert (University of California, San Diego, CA, USA). Constructs encoding small interfering RNAs (siRNAs) directed against p21^CIP1^ were obtained from Invitrogen (Paisley, UK).

### 4.2. Culture of Bovine Testicular Cells

The testicular tissue from a bull calf was cut into 1–3 mm^3^ pieces, and the cells were isolated by enzymatic digestion with 0.25% trypsin–EDTA (Gibco, Grand Island, NY, USA) for 10 min. The cells were cultured in Dulbecco’s Modified Eagle’s Medium (DMEM; Gibco) containing 10 ng/mL human LIF (Sigma, St. Louis, MO, USA) and supplemented with 10% fetal bovine serum (FBS) and antimycotics-/antibiotics (AM−/AB; Gibco). After 2–3 passages, compact colonies were picked and split into other dishes in a 1:3 ratio in the same medium.

### 4.3. Generation of iPSCs

The dissociated testicular cells (5 × 10^5^) were transfected with the *OCT4* gene, as described elsewhere [19,32], using 10 direct-current electrical pulses with an intensity of 20 V at intervals of 50 ms. The cells were placed in 2 mm cuvettes containing 200 μL of DMEM and 10 μg of plasmid DNA, and were treated in an electroporator (CUY 21 Vitro-EX, BEX Co., Ltd., Tokyo, Japan). The cells were then cultured and selected using G418 (100 μg/mL). Two days after selection, the cells were replated onto mitomycin-C-treated mouse embryonic fibroblasts (MEFs) using standard medium supplemented with BMP4 (5 ng/mL; Sigma). The transfected cells were grown in the same medium until iPSCs were detected on day 17. The iPSC colonies were then picked manually and replated onto a new feeder layer (first passage). The bovine iPSCs were then subcultured with trypsin-EDTA treatment, and the medium was replaced every 2 days. The bovine iPSCs (2 × 10^5^) were incubated for 24 h in the presence of the phthalate esters MEHP or DEHP (Sigma-Aldrich) at the indicated doses, and were then harvested.

### 4.4. Stemness Assay and Karyotyping

Alkaline phosphatase activity and immunostaining were assayed as described previously [33]. The antibodies used were directed against: OCT4 (sx-5279; Santa Cruz Biotechnology, Santa Cruz, CA, USA), NANOG (AF1997; R&D Systems, Minneapolis, MN, USA), SOX2 (AB5603; Millipore, Billerica, MA, USA), SSEA-1 (MAB4301; Millipore), and SSEA-4 (MAB4304; Millipore). The fluorescence-labeled secondary antibodies A11034 and A11029 were from Invitrogen (Carlsbad, CA, USA). Nuclei were detected with 0.5 μg/mL of 4′,6-diamidino-2-phenylindole (DAPI, D3571; Invitrogen) for 1 h. Metaphase mitotic chromosomes were prepared using a conventional air-drying technique. GTG staining (G-banding) was performed as described elsewhere [38].

### 4.5. Cell Viability and Apoptosis

The number of viable cells was determined by staining with thiazole orange and propidium iodide (Cell Viability Kit; Becton Dickinson and Company, BD Biosciences, San Jose, CA, USA), according to the manufacturer’s protocols. To differentiate apoptosis from cell necrosis, the cells were stained with fluorescein isothiocyanate (FITC)-labeled annexin V to label apoptotic cells, and with propidium iodide to label permeable cells (FITC Annexin V Apoptosis Detection Kit II; BD Biosciences), and distinguished using flow cytometry. The percentages of apoptotic and necrotic cells were determined using the Bioluminescent Cell Viability Kit II (ADP/ATP; PromoCell GmbH, Heidelberg, Germany). A caspase-3 assay was performed as described elsewhere [19,39].

### 4.6. RNA Extraction and Quantitative Polymerase Chain Reaction (qPCR)

RNA was extracted from cells in the presence of the indicated amounts of MEHP or dimethyl sulfoxide (DMSO), as described elsewhere [40]. The RNA was purified with an RNeasy Mini Kit (2074104; Qiagen, Hilden, Germany), and reverse transcription (RT) was performed with Superscript III reverse transcriptase (18080-093; Invitrogen) and specific primers (Table 2). PCR was performed using the GoTaq^®^ Green Master Mix (M7122; Promega Corp.). To avoid contamination by feeder cells, we chose primer pairs that do not amplify mouse transcripts. Real-time quantitative RT-qPCR (qRT-PCR) was performed on a PRISM™ 7700 system (Amersham Biosystems, Foster City, CA, USA), as described elsewhere [19]. We designed all primers using the public-domain Primer 3 program of the GENETYX-Mac ver.14 software (Hitachi Software, Tokyo, Japan). The primer pairs used are listed in Table 3.

### 4.7. Transfection, Knock down and Luciferase Assay

Bovine iPSCs were transfected with an siRNA against p21^CIP1^ and pGK-CAS-FZD7, or their control vectors (pGK), with 400 ng of total DNA per well in a 24-well plate (5 × 10^4^ cells/well) using 2 μL of the Lipofectamine™ 2000 Transfection Reagent (Invitrogen, Paisley, UK). The cells were cultured in the presence of the indicated amounts of phthalate ester. After 48 h, the levels of p21^CIP1^, FZD7, GSK3β, CTNNB1, LEF1, and TCF3 were measured by qPCR, as described above.

### 4.8. Western Blot Analysis

Cells were lysed in sodium dodecyl sulfate (SDS) lysis buffer (240 mM/L Tris-acetate, 1% SDS, 1% glycerol, 5 mM/L EDTA, pH 8.0) with dithiothreitol, protease inhibitors, and a cocktail of phosphatase inhibitors. The expression levels of proteins were examined using the following antibodies: p21 (C-19: sc-397; Santa Cruz Biotechnology), FZD7 (06-1063; Millipore Co., Billerica, MA, USA), and β-actin (Cell Signaling Technology, Beverly, MA, USA). Anti-rabbit and anti-mouse immunoglobulin (IgG) secondary antibodies were supplied by Invitrogen. The intensities of the bands produced by western blotting were quantified using the GeneTools (Syngene, Cambridge, UK) and Image Lab™ software (Bio-Rad, Hercules, CA, USA). The relative intensities of each band obtained from iPSCs and MEFs were calculated separately by normalizing against that of β-actin. The value of each band obtained from iPSCs was then divided by the value of the corresponding band from MEFs.

### 4.9. Microwestern Arrays

The cells were lysed at the indicated time points and MWAs were performed to measure the protein expression levels and changes, as described previously [19]. The blots were scanned and quantified using a LI-COR Odyssey near-infrared imaging system. β-Actin, and glyceraldehyde-3-phosphate dehydrogenase (Millipore) were used as the loading controls. The intensities of the bands produced by western blotting were quantified using the GeneTools (Syngene) and Image Lab™ software (Bio-Rad). The relative intensity of each band obtained from iPSCs was calculated by normalizing against the corresponding band from MEFs, which was set as 1.0.

### 4.10. Teratoma Formation Assay

Bovine iPSCs (10^6^) in DMEM plus 10% FBS were injected under the kidney capsules of severe combined immunodeficiency (SCID) mice using a 27G needle. The tumors were surgically dissected 6–8 weeks after injection, fixed with 4% formaldehyde, and embedded in paraffin, Subsequently, 4 μm sections were cut and stained with hematoxylin and eosin. The antibodies used were; rabbit anti-human muscle-specific actin (M0635; Dako, Glostrup, Denmark), rabbit anti-human S-100 (N1573; Dako), rabbit anti-human epithelial membrane antigen (EMA, M0613; Dako), and rabbit anti-human cytokeratin (M3515; Dako). Periodic acid Schiff (PAS) staining was performed according to the manufacturer’s specifications (NovaUltra Special Stain Kits; Woodstock, MD, USA).

### 4.11. Statistical Analysis

Differences between the treated and control cells were analyzed with Student’s *t*-test. *p* < 0.05 was considered significant.

## 5. Conclusions

In summary, our results showed that bovine testicular iPSCs are more resistant to AR-dependent apoptosis than testicular cells in response to derivatives of the environmental disruptor MEHP phthalate. MEHP also repressed the expression of AR and inactivated WNT signaling, and then led to the commitment of cells to apoptosis through p21^CIP1^ and the frizzled receptor 7. Testicular iPSCs are useful to screen the toxicants of environmental hormones and maintain the stemness and pluripotency of iPSCs.

## Supplementary Information



## Figures and Tables

**Table 1.** Summary of the characteristics of stemness gene expression and pluripotency. The results of the immunocytochemical analysis of pluripotency and surface markers and RT-PCR analysis of transcripts of the “stemness” genes (encoding OCT4, SOX2, c-MYC, KLF4, STAT3, SUZ12, DNMT1, and MEF2A) in bovine testis cells and iPSCs are summarized.

Characterizationof bovine testicular cells and induced pluripotent stem cells										

Cell type	Teratoma formation	Cell-surface markers			Stem cell genes					
	
		SSEA-1	SSEA-3 SSEA-4	AP	4-Oct	Nanog	Sox2	Klf4	C-Myc	Stat3
Testicular cells	−	+/− a	+/− a	+/− a	−	−	−	+	+	+
Testicular iPSCs	+	+	+	+	+	+	+	+	+	+

a The indicated cell type expressed each cell-surface marker weakly.

iPSCs: Induced pluripotent stem cells.

**Table 2.** Nucleotide sequences of the primers used for stemness-related genes and the expected sizes of the DNA amplified.

Primers	Gene	5′→3′	Size of amplified DNA (bp)
1	oct3/4-F	ccctgaggagtcccaggacat	356
	oct3/4-R	gcaggaacatgctctccaggtt	
2	sox2-F	ctacagcatgatgcaggaccagct	381
	sox2-R	tgctgggacatgtgaagtctgctg	
3	gklf4-F	gttcgtgttgaaggcgtcgctg	173
	gklf4-R	tgcacgaggagacagcctcct	
4	c-myc-F	ccaagctcgtctcggagaagc	334
	c-myc-R	tcagagtcgctactggtcgtgg	
5	SALL4-F	catagacaaggccaccaccgacc	276
	SALL4-R	atgtgcatgcggatgtgctgct	
6	ID1-F	acgacatgaacggctgctactc	142
	ID1-R	tgggattccgagttgagctccaa	
7	EED-F	atagcaatacaagccatcccctgc	223
	EED-R	aatattgccaccagagtgtccgtc	
8	SUZ12-F	gcagttcactcttcgttggacagg	449
	SUZ12-R	cctgaggatttcctgcataggagc	
9	STAT3-F	gtctaacaatggcagcctctcagc	405
	STAT3-R	aagagtttctccgccagcgtc	
10	GADD45A-F	ctttggaggaattctcggctggag	252
	GADD45A-R	cattctcacagcagaatgcctgg	
11	SMAD4-F	ttcatgactttgagggacagcca	438
	SMAD4-R	gctcattgtgaactggtggccag	
12	DNMT1-F	cggtgttcacaaaggactgcaacg	359
	DNMT1-R	gtactgaccagcctgcagcac	
13	DNMT3A-F	tgcaagaactgcttcctggaatgc	398
	DNMT3A-R	accagaagccctgtagcaattcc	
14	TERT-F	cctacgtggtggagctgctcag	155
	TERT-R	tgacagttctcgaagccgcac	
15	MEF2A-F	atgcctccactgaatacccaaagg	217
	MEF2A-R	acacctgtcccagagacagcat	
16	MEF2C-F	ggtatggcaatccccgaaactcac	408
	MEF2C-R	gccagccagttactgacccaagat	

**Table 3.** Nucleotide sequences of the primers used for quantitative PCR (qPCR).

Primers	Gene	5′→3′
1	Androgen receptor-F	CAGTGGATGGGCTGAAAAAT
	Androgen receptor-R	AGGAGCTTGGTGAGCTGGTA
2	p21/Cip1-F	ATGGGTCTGGGAGATGTGAG
	p21/Cip1-R	CATATGGGAGCCAGGAGAAA
3	GSK3β-F	CGTGATCCTTCCGCCGCTTCC
	GSK3β-R	TCCACTCCCTTTCCTTGGAGGGCA
4	CTNNB1-F	CCATTCCATTGTTTGTGCAG
	CTNNB1-R	TGCATATGTTGCCACACCTT
5	Frizzled	7-F TTGCCTCTGGACCTTTGCAC
	Frizzled	7-R CGTGGTCTGGCACTGAGATG
6	LEF-F	CACCCTGAAGAGGAAGGTGAC
	LEF-R	GAGGTTTGTGCTTGTCTGGC
7	TCF3-F	CGTGGCAGCTGATACAGCCGA
	TCF3-R	GGCCGCTTTAGGGTTCAGGTTACG
8	GAPDH-F	GGGTCATCATCTCTGCACCT
	GAPDH-R	GGTCATAAGTCCCTCCACGA

## Abbreviations

AR: androgen receptor
DEHP: di-(2-ethylhexyl) phthalate
DMSO: dimethyl sulfoxide
EDCs: endocrine-disrupting chemicals
FZD7: Frizzled receptor 7
iPSCs: induced pluripotent stem cells
MEFs: mouse embryonic fibroblasts
MEHP: mono-(2-ethylhexyl) phthalate
OCT4: octamer-binding transcription factor 4
p21^CIP1^: cycling-dependent kinase inhibitor 1
qPCR: quantitative RT–PCR
RT–PCR: reverse transcription polymerase chain reaction
