# Supplementary material for: Bovine Induced Pluripotent Stem Cells Are More Resistant to Apoptosis than Testicular Cells in Response to Mono-(2-ethylhexyl) Phthalate
Source: Int J Mol Sci. 2014 Mar 20;15(3):5011–31. doi: 10.3390/ijms15035011 (PMC3975437; doi:10.3390/ijms15035011)
Supplement: Supplementary file 1 [file ijms-15-05011-s001.pdf]

Supplementary Information

Figure S1. Transfection efficiency of bovine testicular cells using electroporation. The efficiency of transfection of bovine testicular cells by electroporator with the pEGFP expression vector was examined with various voltage pulses, including at 10 V (**a**); 20 V (**b**); and 30 V (**c**).

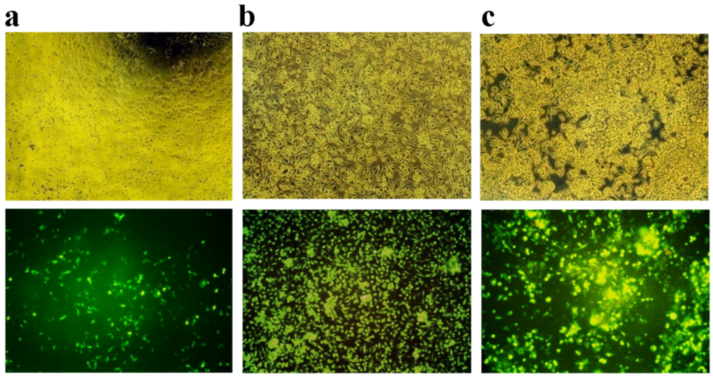

Table S1. Summary of transfection efficiency. The transfection was calculated as the number of transfected cells/number of surviving cells. The numbers of surviving cells were estimated with trypan blue staining.

Examination of transfection efficiency of bovine testicular cells by electroporator with EGFP expression vector

Voltage (V)	Pulse interval (ms)	Survival rate (%)	Transfection rate * (%)
10	50	57.0 ± 9.4	30.0 ± 1.3
20	50	63.3 ± 4.7	66.7 ± 4.7
30	50	56.7 ± 9.4	53.3 ± 12.5

*: No. of transfected cells/No. of survived cells.

Figure S2. Effects of the forced expression of the frizzled receptor, FZD7, on the expression of genes related to WNT/β-catenin signaling pathway in testicular cells and iPSCs. Relative expression of genes encoding Frizzled 7 (**a**); GSK3β (**b**); CTNNB1 (**c**); LEF1 (**d**); and TCF3 (**e**) in bovine testicular cells and iPSCs as indicated. (1, control DMSO (0.001%); +, 10^{-4} M MEHP in the presence (+) or absence (−) of the forced expression of pGK-CAS-FZD7 or its control plasmid pGK). The values were expressed as means ± SEM, $n \geq 3$, ** $p < 0.01$.

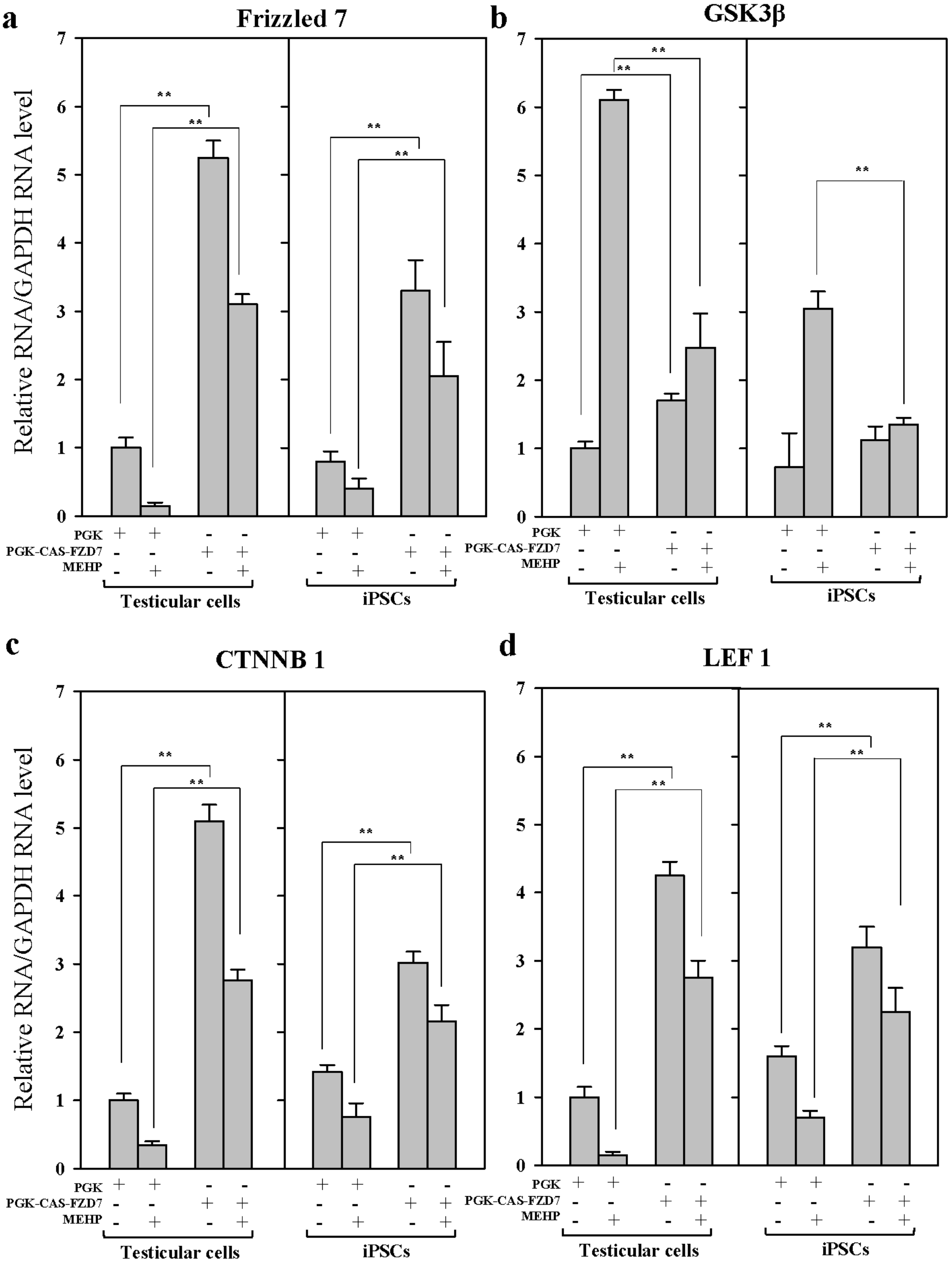

Figure S2. *Cont.*

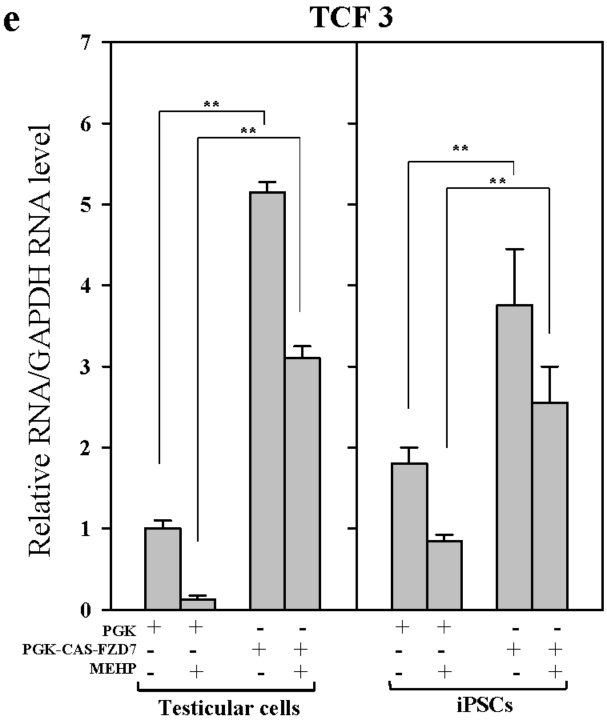

© 2014 by the authors; licensee MDPI, Basel, Switzerland. This article is an open access article distributed under the terms and conditions of the Creative Commons Attribution license (http://creativecommons.org/licenses/by/3.0/).
